# Large Area Nanoparticle Alignment by Chemical Lift-Off Lithography

**DOI:** 10.3390/nano8020071

**Published:** 2018-01-27

**Authors:** Chong-You Chen, Chia-Hsuan Chang, Chang-Ming Wang, Yi-Jing Li, Hsiao-Yuan Chu, Hong-Hseng Chan, Yu-Wei Huang, Wei-Ssu Liao

**Affiliations:** Department of Chemistry, National Taiwan University, Taipei 10617, Taiwan; d03223108@ntu.edu.tw (C.-Y.C.); r03223126@ntu.edu.tw (C.-H.C.); d03223129@ntu.edu.tw (C.-M.W.); r03223170@ntu.edu.tw (Y.-J.L.); r04223102@ntu.edu.tw (H.-Y.C.); r02223161@ntu.edu.tw (H.-H.C.); r03223167@ntu.edu.tw (Y.-W.H.)

**Keywords:** nanoparticle, chemical lift-off lithography, self-assembled monolayer, patterning, selective deposition

## Abstract

Nanoparticle alignment on the substrate attracts considerable attention due to its wide application in different fields, such as mechanical control, small size electronics, bio/chemical sensing, molecular manipulation, and energy harvesting. However, precise nanoparticle positioning and deposition control with high fidelity are still challenging. Herein, a straightforward strategy for high quality nanoparticle-alignment by chemical lift-off lithography (CLL) is demonstrated. This technique creates high resolution self-assembled monolayer (SAM) chemical patterns on gold substrates, enabling nanoparticle-selective deposition and precise alignment. The fabricated nanoparticle arrangement geometries and dimensions are well-controllable in a large area. With proper nanoparticle surface functionality control and adequate substrate molecular manipulation, well-defined nanoparticle arrays with single-particle-wide alignment resolution are achieved.

Nanoparticle arrangement on a supporting substrate provides a variety of interesting applications, not only in fundamental scientific understanding, but also produced platform property engineering [1,2,3]. For example, arrays of nanoparticle arrangement in different geometries can benefit the development of plasmonic devices for more efficient optical biosensing, molecular spectroscopy, and light manipulation applications [4,5]. However, selective and precise particle positioning encounters obstacles such as nonspecific adhesion and difficult fabrication. Two main categories of operation strategies were performed including physical geometry-induced particle stacking and interface molecular force-driven matter anchoring. The use of substrate physical geometry on alignment operation depends on capillary force-induced particle movement toward template edges when carrying solvents evaporate. These strategies can be applied to create diverse particle array geometries with tunable pattern sizes [6,7,8,9,10,11,12,13]. However, the initial template fabrication process restricts the following particle placement, especially when geometries with very small dimensions are requested. Besides, the lack of nonspecific adhesion control disrupts the integrity of the fabricated particle array, which may induce serious interferences in subsequent applications. On the other hand, utilization of interface molecular force control takes advantages of specific interactions between nanoparticles and supporting substrates. With well-tunable surface functionalities, large area particle arrays can also be accomplished on a comparably flat substrate without the need for preliminary template fabrication [14,15,16,17]. However, precise pattern geometry control, rendering both high resolution and minimized nonspecific particle adhesion properties, are still very challenging. This is due to the difficulties in creating sharp edge molecular patterns in a convenient route with high feature stability. The situation further escalates when a very narrow molecular gap is required to position targets down to the single particle scale, which is extremely difficult to control with conventional fabrication processes. Furthermore, appropriate modulation for interfaces interaction is necessary to achieve minimized nonspecific particle adhesion, but a simple operation protocol for this purpose is still arduous.

Chemical lift-off lithography (CLL) creates sub-30 nm resolution molecular patterns by a straightforward self-assembled monolayer (SAM) top-layer Au-thiolates rupturing process [18,19,20,21,22]. This lift-off operation not only generates freshly exposed Au regions with residual alkanethiol molecules, but also creates atomic scale step-edges at the molecular pattern boundary in a one-step fashion. The fundamental idea behind this technique lies on initiating reactive moieties on the feature-carrying soft material-based polydimethylsiloxane (PDMS) stamp to interact with specific corresponding functional groups on a SAM pre-modified Au substrate. The strong interface interaction induces surface Au–Au bond breakage when the conformal sealed PDMS stamp is separated from the SAM-modified Au substrate. An abundance of active hydrophilic functional groups, e.g., hydroxyl and amine groups, have been tested with this operation when an oxygen plasma is used to introduce active hydroxyl moieties on the PDMS side for condensation reactions [18,23]. It is found that distinct molecular patterns are created in this straightforward contact-and-lift operation as long as the interface reactions can be initiated. Notably, this contact-induced reaction is very selective and the produced post-lift off regions are available for the back-insertion of various molecules and probes [19,24]. Multiplexed molecular platforms can therefore be fabricated via surface environment manipulation and has been effectively applied in numerous fields, such as bio-capturing and chemical sensing [19,24,25,26,27]. Due to high achievable resolution, robust operation, and sufficient integrity properties of the technique, we realized that the CLL-reated molecular patterns are highly potent in providing precise guidance for aligning nanoparticles in large areas. To achieve this perspective application, molecular functionality selection is also crucial in reducing nonspecific particle adhesion between two interfaces. Through sufficient attractive force between target particles and desired allocating regions, in addition to repulsion stress from adjacent areas, precise alignment with minimum nonspecific deposition is expected.

Our strategy to achieve large area nanoparticle alignment utilizing chemical lift-off lithography is demonstrated in Figure 1. A hydroxyl-terminated 11-mercaptoundecanol (MCU) SAM-modified Au substrate is first conformal sealed with an oxygen-plasma treated PDMS stamp. The separation of stamp from Au substrate creates adjacent regions with distinct properties, i.e., post-lift off Au-exposing regions and those covered by the original SAM. It is expected that the functionality of post-lift off regions should be very different from the original hydroxyl-terminated MCU SAM-modified areas, which provides an opportunity to differentiate the surface property of a depositing nanoparticle. A citrate-capped Au nanoparticle suspension solution is therefore introduced onto this substrate to test the selectivity of created regions toward specific particle surface functionality. As can be seen Figure 1, these particles deposit onto the hydroxyl-terminated MCU SAM-modified regions with a high selectivity, while a very low number of nonspecifically adhered particle is found in the post-CLL region. We attribute this selective nanoparticle deposition to hydrogen bonding between particle surface coated citrate molecules and hydroxyl terminal groups of the SAM. To verify this hydrogen bonding-induced selective deposition phenomenon, some examination are applied as shown in Figure 2. It is found that a great number of deposited Au nanoparticles on the hydroxyl-terminated MCU SAM are removed when hot water or heated urea are used to clean the surface. This finding leads us to believe hydrogen bonding between nanoparticles and the supporting substrate is sufficient to induce this obvious particle attraction. It is also important to note that very little nonspecific particle adhesion happens in the post-lift off region. The previously reported investigation of this post-lift off area points to ~30% alkanethiol residuals remaining inside this matrix under this operation condition [18,24,27,28,29]. These molecules are therefore exposing their alkane chains to the outside environment, which is expected to increase the substrate hydrophobicity. A series of water contact angle studies are employed to investigate the hydrophobicity change of hydroxyl-terminated MCU SAM-covered Au along with the CLL treating time. As shown in Figure S1, the water contact angle increases when the substrate is treated by an activated stamp for a longer duration until reaching a steady plateau. Longer stamp seal time is expected to increase the number of removed hydrophilic alkanethiol molecules, whereas the hydrophobic alkane chains of the randomly distributed residual molecules are exposed to the outside environment. Integrated with a raised amount of exposed fresh Au areas, these factors compensate each other and give a maximum level of water contact angle at the longer stamp seal time. Under increased hydrophobicity and reduced hydroxyl-terminated alkanethiol molecules in the post-lift off region, a low amount nonspecific adhesion of citrate-capped Au nanoparticle is expected. The CLL-created distinct molecular regions are therefore able to selectively anchor introduced nanoparticles at specific positions.

To achieve well-controllable selective nanoparticle deposition, important experimental factors are introduced to modulate surface anchoring nanoparticle density. As demonstrated in Figure 3A and Figure S2A, higher quantities of Au nanoparticles are anchored on the hydroxyl-terminated MCU SAM-modified substrate with increasing particle suspension solution concentration. In contrast, no obvious particle adhesion is observed on the CLL-treated substrate even under application of highly concentrated particle solutions. Furthermore, the nanoparticle and substrate incubation time can also be used to modulate surface anchoring particle quantities. As shown in Figure 3B and Figure S2B, longer particle solution deposition time results in higher surface nanoparticle densities. Similarly, minimum nonspecific particle adhesion on the CLL-treated substrate is also observed under these operations. It is therefore concluded that the substrate anchoring nanoparticle quantities are well-tunable via appropriate surface functionality adjustment and straightforward CLL-induced molecular environment manipulation.

Relying on this interface interaction guidance and precise molecular environment control, large area nanoparticle arrangement in desired regions can be achieved. As demonstrated in Figure 4, nanoparticle arrays with different geometries in diverse dimensions are produced. The citrate-capped Au nanoparticles selectively deposit on hydroxyl-terminated MCU SAM-modified areas through hydrogen-bonding interaction. On the other hand, post-lift off regions resist the nanoparticles’ nonspecific adhesion and result in distinct particle arrangement boundaries (the off-pattern particles are results of dust disturbance in stamp-substrate contact areas due to experiment ambient operations without the use of clean-room facilities). Moreover, necklace-like nanoparticle alignments are also achieved when minuscule gap patterns are created by CLL. This smaller-than-nanoparticle molecular gap pattern enables the single-nanoparticle-wide alignment on substrates. It is important to note that only few existing techniques are able to achieve very high resolution molecular patterns that are able to guide the deposition of nanoparticles with straightforward processes and high array integrity. Taking advantage of one-step operation induced molecular environment changes via the chemical lift-off process, large area robust surface molecular patterns are created to achieve single-nanoparticle-wide positioning resolution.

In summary, chemical lift-off lithography opens a straightforward route to create very precise nanoparticle alignment and selective positioning on the supporting substrate. The strategy utilizes activated soft material rubber stamp to initiate a contact-induced reaction between polymer surface moieties and substrate SAM tail groups. Following the stamp separation process induces interface Au–Au bond breakage, leading to the creation of freshly exposed Au areas with some residual alkanethiol molecules. This unique molecular environment resists citrate-capped Au nanoparticle nonspecific adhesion due to highly reduced number of hydrophilic alkanethiols and increased residual molecule exposing hydrophobic alkane chains. The nanoparticles are consequently deposited onto hydroxyl-terminated SAM covered areas, where hydrogen-bonding plays a key role in attracting citrate-capped Au nanoparticles. This approach can therefore be applied to fabricate nanoparticle arrays with different geometries and dimensions with clear arrangement boundaries. Under appropriate deposition conditions, spatially precise nanoparticle alignment with single-particle-wide resolution in a large area is achieved with this approach. We envision this technique to be able to connect with nanomaterial-based devices and its operation convenience should provide relevant applications a straightforward fabrication route.

## Figures and Tables

**Figure 1:**
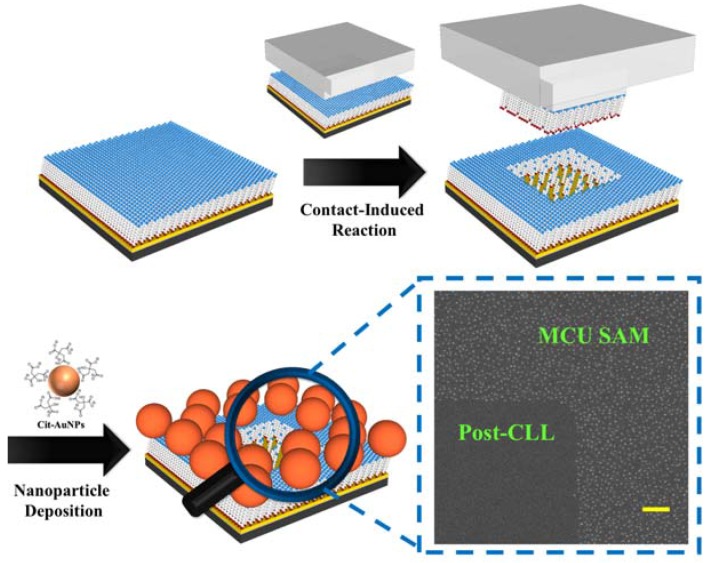
Schematic illustration of citrate-capped Au nanoparticle selective deposition on the chemical lift-off lithography (CLL)-created molecular pattern. The hydroxyl-terminated mercaptoundecanol (MCU) self-assembled monolayer (SAM) is used as the operation matrix. In the CLL process, a polydimethylsiloxane (PDMS) stamp is first treated with oxygen plasma for 40 s, and then conformal sealed onto a MCU SAM-modified substrate for 24 h. After separating stamp and substrate, 100 pM Au nanoparticle suspension solution is dropped onto the substrate for 1 h of incubation. Finally, the substrate is wash with deionized (DI) water and blown dried with nitrogen gas, producing clear nanoparticle selective deposition showing in the scanning electron microscope (SEM) image. The scale bar is 1 μm.

**Figure 2:**
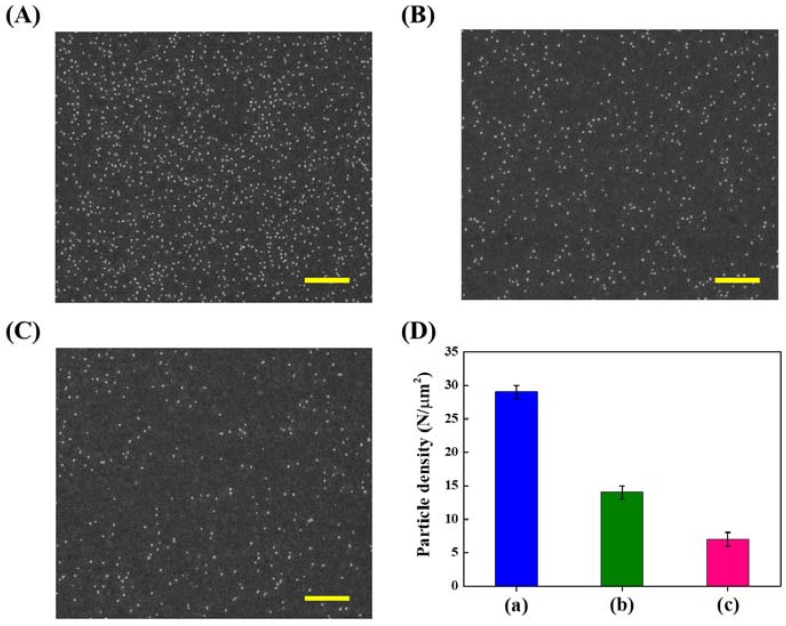
SEM images of Au nanoparticles remaining on the MCU SAM-modified Au substrate after different treatments: (**A**) Without any cleaning; (**B**) 30 min of 80 °C hot water cleaning; and (**C**) 30 min of 60 °C 0.1 M urea cleaning. (**D**) Particle densities counted from (**A**–**C**), where (a), (b), and (c) are histogram columns corresponding to (**A**), (**B**), and (**C**), respectively. The scale bars are 1 μm. (*N* = 3).

**Figure 3:**
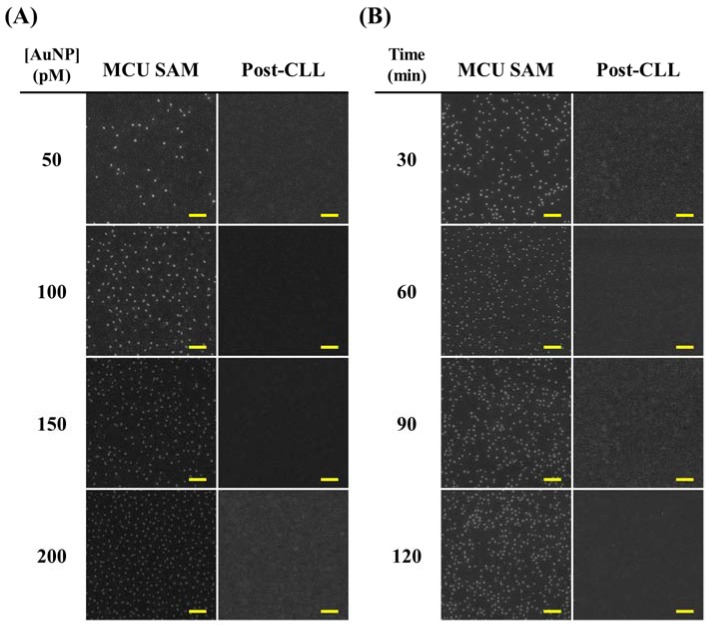
SEM images showing different Au nanoparticle densities on the CLL-treated substrates. The CLL process is operated under the 24 h stamp seal time condition. (**A**) 1 h of nanoparticle deposition time is fixed with changing solution concentrations; (**B**) 150 pM of nanoparticle suspension solution is used with various deposition time. The scale bars are 2 μm.

**Figure 4:**
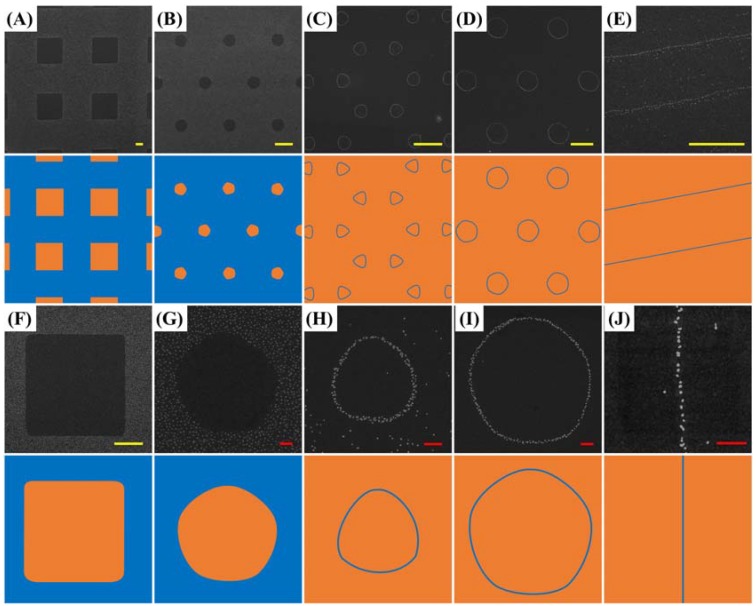
Au nanoparticle arrays with different geometries and dimensions created by CLL-induced selective particle alignment. (**A**–**E**) are large area SEM images with their corresponding zoom-in images showing in (**F**–**J**). The corresponding cartoon images represent the stamp-substrate contact areas (orange) and noncontact regions (blue) during the conformal sealing procedure. The CLL process is operated under conditions of 24 h stamp seal time and 1 h of 100 pM nanoparticle deposition duration. The yellow and red scale bars are 5 and 0.5 μm, respectively.

## Supplementary Materials

The following are available online at http://www.mdpi.com/2079-4991/8/2/71/s1, Figure S1: Water contact angle measurements of MCU SAM-modified Au substrates under different stamp seal time treatments in the CLL process. (*N* = 3); Figure S2: Au nanoparticle densities on the CLL-treated substrates corresponding to SEM images shown in Figure 3. The CLL process is operated under the 24 h stamp seal time condition. (A) 1 h of nanoparticle deposition time is fixed with changing solution concentrations. (*N* = 4); (B) 150 pM of nanoparticle suspension solution is used with various deposition times. (*N* = 4).
