# Supplementary material for: Large Area Nanoparticle Alignment by Chemical Lift-Off Lithography
Source: Nanomaterials (Basel). 2018 Jan 27;8(2):71. doi: 10.3390/nano8020071 (PMC5853703; doi:10.3390/nano8020071)
Supplement: Supplementary file 1 [file nanomaterials-08-00071-s001.pdf]

Communication

Large Area Nanoparticle Alignment by Chemical Lift-Off Lithography

Chong-You Chen †, Chia-Hsuan Chang †, Chang-Ming Wang, Yi-Jing Li, Hsiao-Yuan Chu, Hong-Hseng Chan, Yu-Wei Huang and Wei-Ssu Liao *

Department of Chemistry, National Taiwan University, Taipei 10617, Taiwan; d03223108@ntu.edu.tw (C.-Y.C.); r03223126@ntu.edu.tw (C.-H.C.); d03223129@ntu.edu.tw (C.-M.W.); r03223170@ntu.edu.tw (Y.-J.L.); r04223102@ntu.edu.tw (H.-Y.C.); r02223161@ntu.edu.tw (H.-H.C.); r03223167@ntu.edu.tw (Y.-W.H.)

* Correspondence: wsliaochem@ntu.edu.tw; Tel.: +886-2-3366-8712

† These authors contributed equally to this work.

Experimental Materials

11-mercaptoundecanol (MCU), sodium citrate, tetrachloroauric acid, and hexamethyldisilazane (HMDS) were purchased from Sigma-Aldrich (St. Louis, MO, USA). Positive photoresist AZ6112 was purchased from AZ Electronic Materials Taiwan Co., Ltd. (Taipei, Taiwan). T238 developer was purchased from Control Chemitech Inc. (Taoyuan, Taiwan). Silicon wafers were obtained from Mustec Corp. (Hsinchu, Taiwan). SYLGARD 184 silicone elastomer base and curing agent were purchased from Dow Corning Corp. (Midland, MI, USA). DI water (>18 MΩ·cm) was obtained from the ELGA PURELAB classic system (Taipei, Taiwan).

Instruments.

SEM images were collected by the JSM-6700F field emission scanning electron microscope (JEOL Ltd., Tokyo, Japan). Water contact angle measurements were performed using the OCA 15EC contact angle analysis system (DataPhysics Instruments GmbH, Filderstadt, Germany).

Preparation of Citrate-Capped Au Nanoparticles.

Au nanoparticles with 45 nm diameter were synthesized by citrate reduction of tetrachloroauric acid [1]. 0.5 mL of 1 % (*v*/*v*) sodium citrate aqueous solution was quickly added to a 50 mL 0.01 % (*v*/*v*) tetrachloroauric acid aqueous solution in a reflux system with stirring. The solution was kept refluxing until the color changed from pale yellow to wine red. After 8 min of reaction, the solution was quickly cooled down in an ice bath. The resulting Au nanoparticle solution was stored at 4 °C in the dark before usage.

Nanoparticle alignments by CLL.

Silicon substrates with 100 nm-thick Au and 5 nm-thick Cr adhesive layer were prepared by thermal evaporation. Au substrates were immersed in the 0.5 mM MCU ethanoic solution for over 6 h to form self-assembled monolayers. After MCU SAM formation, the substrates were washed by ethanol to remove excess MCU molecules, and blown dry with nitrogen gas. PDMS stamps with various patterns were fabricated by standard photolithography-created master molds. These masters were generated via spin-coating 1.5 μm-thick AZ6112 photoresist onto a 2 in. silicon wafer, pre-baked at 120 °C for 5 min, exposed to UV light along with a photomask (30 mW/cm^2 for 1 s), immersed in T238 developer for 45 s, and post-baked at 120°C overnight. A 10:1 mass ratio of SYLGARD 184 silicone elastomer base and curing agent was thoroughly mixed, degassed under vacuum, cast onto master molds, and cured on an aluminum-top hot plate at 100 °C overnight. The PDMS stamps were separated from the master molds, sequentially rinsed by acetone and isopropanol, and then blown

dry by nitrogen gas. The prepared stamps were activated by 40 s exposure to oxygen plasma (Harrick Plasma PDC-32G, Ithaca, NY, USA) at a power of 18 W with 0.5 mbar oxygen flow. Thereafter, the stamps were conformal sealed onto the MCU SAM-modified substrates for a various amount of times. After separating contact-sealed stamps from Au substrates, citrate-capped Au nanoparticle solutions were quickly dropped onto Au substrates at 35 °C. After typically 2 h of incubation, the substrates were gently rinsed by DI water, blown dry with nitrogen gas, and stored at 4°C in dark before characterization.

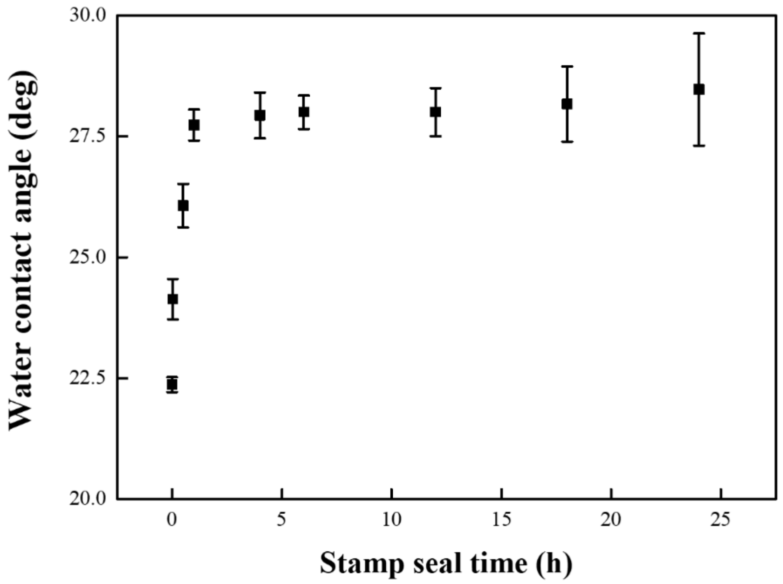

Figure S1. Water contact angle measurements of MCU SAM-modified Au substrates under different stamp seal time treatments in the CLL process. ($N = 3$).

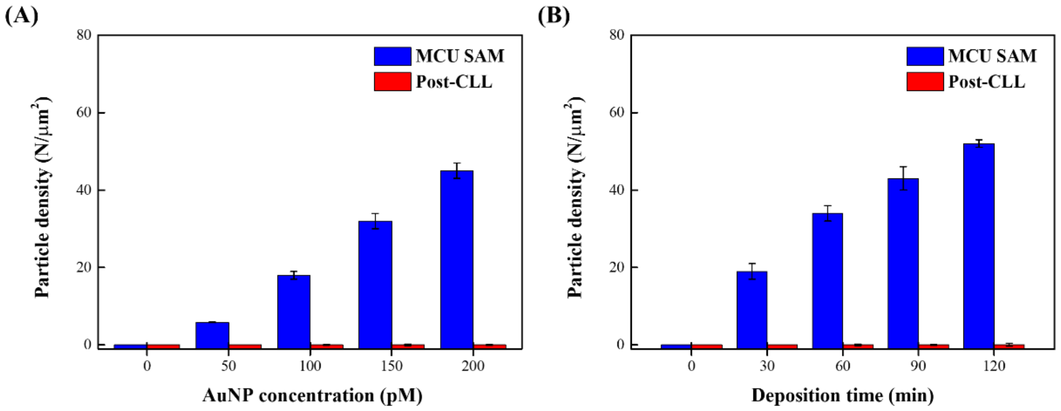

Figure S2. Au nanoparticle densities on the CLL-treated substrates corresponding to SEM images shown in Figure 3. The CLL process is operated under the 24 h stamp seal time condition. (**A**) 1 h of nanoparticle deposition time is fixed with changing solution concentrations. ($N = 4$); (**B**) 150 pM of nanoparticle suspension solution is used with various deposition times. ($N = 4$).

Reference:

1. Frens, G., Controlled Nucleation for the Regulation of the Particle Size in Monodisperse Gold Suspensions. *Nature Phys. Sci.* **1973**, *241*, 20-22.
